# The Principles of Stock-Breeding

**Published:** 1901-04

**Authors:** George B. Jobson

**Affiliations:** Franklin, Pa.


### THE PRINCIPLES OF STOCK-BREEDING.

By George B. Jobson, V.S.,
FRANKLIN, PA.

Although the origin of the various races of domestic animals is veiled in obscurity, and we know not at what remote period or by whom their wild progenitors were first brought under the dominion of man, yet it is reasonable to suppose that the breeding of these tamed animals when first domesticated must have been coeval with primal civilization. In fact, their subjugation and subservience to man's uses must have been one of the main factors in his advance from a state of barbarism to a state of civilization. The existence of the horse, ox, and sheep can be traced back to prehistoric times, their bones having been found in the lake dwellings of Switzerland. In Genesis, Abel is called a keeper of sheep, while the horse, ox, and ass are frequently mentioned in Bible history, and we are told that the wealth of the patriarchs, these nomads of Mesopotamia and Syria, consisted in the multitude of their flocks and herds. The Assyrian tablets and the paintings in the Egyptian tombs invariably portray the horse employed for purposes of pomp, for hunting, or in war-like operations; in the latter country, which in the time of the Pharaohs was the granary of the civilized world, the ox is represented as being largely used in agriculture. These pictured animals do not differ materially in form from the races of domestic animals existing at the present time.

The art of breeding resolves itself into two branches: its principles and its practice. Unless the former are correct, the latter will be continually at fault, and it will be a matter of chance or uncertainty whether success or failure results from the union. When certain effects resulting from mating animals are viewed not as matters of chance, but as the result of certain definite principles relating to the perpetuation and improvement of breeds, the systematic application of which is clearly under the control of man, then breeding will be successful and profitable. It has been stated and accepted as an axiom that eminent breeders are born and not made. It is true eminent pioneers in the art of breeding, such as Bakewell and the Brothers Colling, appear instinctively to have grasped at those forms best calculated to improve, and lay the foundation of the respective breeds of sheep and cattle, with which their names are so intimately associated, yet they also must have been governed by certain principles, patient adherence to which enabled Bakewell to create the New Leicester sheep, and Robert and Charles Colling to lay the foundation of the improved Shorthorn.

The art of breeding always implies that the breeder has in his mind an ideal form or model after which he attempts to mould his strain. Guided by an eye educated by long experience he detects those slight differences of form which verge toward and those which depart from his ideal. In the selection of individuals for pairing—generally speaking, those animals having certain points peculiarly well developed—are mated with those excelling in other directions, in order that a harmonious whole may follow the union. In breeds of the domesticated animals designed for the butcher, a disposition to accumulate fat readily and mature early are the qualities desired. Such animals assimilate food more profitably than those whose conformation indicate qualities of an opposite nature. They have the capacity to convert a given amount of food into the largest amount of meat and the development of that meat on those parts of the body most esteemed for the table. Early maturity is especially to be desired for two reasons: first, the younger the animal the more rapid the increase in weight from a given quantity of food consumed; and, second, there is an earlier return on capital invested. In a general way, these qualities of good form and early maturity are indicated by a parallelogrammic body, with straight upper and lower lines, well-sprung ribs and broad loins, small head, short neck and legs, with small bones, the whole carcass when matured being smoothly covered with a good coating of flesh. In cattle the hide ought to be moderately thick, soft, mellow, and elastic. Combined with these good qualities, the disposition ought to be placid and phlegmatic, showing a tendency to lay on fat.

In dairy breeds, while a few of the foregoing qualities are desirable, the disposition to lay on fat must be subordinate to the development of the lactic system. The capacity to give a large amount of milk is generally indicated by a small, lean head, moderately long, thin neck, shoulders thin and sloping, brisket and fore-quarters light, fine withers, ribs well rounded, abdominal cavity large, fully developed, and deep at the flank, loin broad, and hips wide, the hind legs standing well apart, allowing plenty of space for the udder, which ought to be roomy behind, running up toward the vulva, and well forward along the belly. The teats ought to be of good size and set well apart, the texture of the udder being pliable and thin. Skin moderately thick, soft, and mellow, covered with fine mossy hair.

A well-bred steer and a well-bred horse express qualities of an entirely opposite character, the use for which each animal is intended being entirely different. While great muscular activity and vigor are necessary in all horses, a large powerfully built frame covered with strong muscles, having a certain amount of adipose tissue combined with large bone, are essential in dray horses in giving the necessary weight for pulling heavy loads, the disposition being quieter and more phlegmatic than in horses intended for speed. Thoroughbred horses have a smaller and denser bone, and when in condition, muscles hard, firm, and free from fat, tendons clean, large, and strong, standing well out from the canon bones, chest deep and roomy, and, in combination with this physical development, a large amount of nervous energy, giving the stoutness and endurance so necessary in the race horse.

*Heredity.* "Like begets like" is the old adage of the breeder, and on this principle breeds are formed. As we have become better acquainted with the laws governing breeding, we have added to this axiom "or the likeness of an ancestor." The transmission of certain peculiarities is the essential characteristic of a breed; on the other hand, this is not absolutely true, for were there an unbroken uniformity in the character of a type in each successive generation the formation of new breeds would be impossible. Environment has a strong influence on the character of a breed. As long as the conditions of life remain the same, we have reason to believe that a modification once inherited and firmly fixed may continue inherited for an indefinite number of generations. On the other hand, we have evidence that variability when once it has come into play does not cease under domestication for a long period. The silent influences of soil and climate exert a far more powerful influence on the frame and constitution than is generally imagined, and certain characters latent in a breed will under favorable conditions be developed. On the contrary, the initial impetus given to a breed is not always carried out in consequence of the surroundings being unfavorable. The transmission of hereditary qualities and variability, two apparently antagonistic principles, render the improvement of old and the formation of new breeds possible. The three leading principles relating to breeding are: heredity, variability, and selection. Unless valuable qualities are possessed by the parents it will be useless to expect them in the offspring. Generally speaking, breeders act on the principle of selecting the best individuals in each generation and pairing them. Defects are as easy of transmission, if not more so, than good qualities, and not merely grave physical defects, but mental ones as well. Horses frequently inherit ringbones, spavins, or curbs from an ancestor. Some families are termed "quitters," being deficient in gameness. Cows of good family sometimes have poorly developed udders, and some bulls have vicious tempers, all traceable to inheritance.

*Variation.* Darwin says: "Species are not immutable productions." Our domesticated animals are largely influenced by physical conditions of life, such as amount and quality of food and shelter, and having a more elastic organization, modification is more easily effected than in wild animals. In wild animals, the conditions of life having been unchanged for ages, the power of heredity is strong; consequently in a state of nature there is less variation in any one species than under domestication. Modification in size is caused by the amount and quality of food, the heavy Shire horse, the Lincoln sheep, and the heavy breeds of cattle are native to the rich midland counties of England. Thickness of skin and length of hair are influenced by climate and exposure; for example, the Highland cattle of Scotland and the Shetland pony. Color by climate, the fauna of the polar regions being almost uniformly white.

Man can effect nothing by way of improvement until some of his stock begin to vary in the right direction. The breeder selects a congenital variation which suits his requirements, and by breeding from the animal exhibiting that variation obtains a new or improves an old breed. Variation may take the form of degeneration, and without proper food and care a breed will gradually degenerate and run out; therefore, good food and shelter are absolutely necessary in improving a breed.

Ancestral peculiarities, remote it may be, give rise to variations and the possibility of characters existing in a latent condition is of the utmost importance to the breeder since upon it depends the danger of atavism or reversion. This is a serious hindrance to the breeder, and undesirable characters supposed to be eliminated will after several generations crop out even with his most scrupulous care. The more highly developed any particular quality in a breed the more liable is it to variation. There may be truly said to be a constant struggle going on between the tendency to reversion to a less perfect state as well as a tendency to form new variations.

*Selection.* The key to the origin and improvement of breeds is man's power of accumulative selection. In this way the distinctive characters of each breed have been formed. Nature gives successive variations, man adds them up in certain directions useful to himself. On the other hand, nature selects these variations in form which are to the advantage of the individual, and which in the struggle for existence lead to the survival of the fittest. This principle of selection by man is not hypothetical. Bakewell may be said to have made the new Leicester sheep, and the Brothers Colling the Shorthorn breed of cattle, previously termed Teeswaters or Durhams. Youatt speaks of the principle of selection as "that which enables the agriculturist not only to modify the character of his flock, but to change it altogether. It is the magician's wand by which he may summon into life whatever form and mould he pleases." While this may appear overdrawn, it is a fact that the organization of our domestic animals is comparatively plastic; and to improve or change the character of a breed, rigid and methodical selection with a definite object in view is necessary to secure and fix the desired qualities. It is the work of a lifetime, and patient work and indomitable perseverance are necessary to succeed. While a good pedigree is essential an animal, no matter how well bred, which is deficient in good form or those distinctive qualifications rendering this particular family desirable ought to be rejected for breeding as a degenerate variation from the true type of the family. By selection and careful training English race horses have come to surpass in fleetness and size the parent Arab from which they are descended. The sheep and cattle of England by the same method have increased in size and early maturity. By selection our American trotters and pacers as harness horses surpass in speed any other race in the world, and by selection our American Jerseys are larger, more robust, and give a larger amount of milk and butter than their ancestors on the island.

*In-breeding.* Much has been said in favor of and against in-breeding. Judiciously used by rigidly selecting the best animals for breeding purposes, it is a powerful factor for increasing the prepotency of the strain in the hands of the experienced breeder. Every new breed must originate in a few individuals possessing some special peculiarities. Nearly related individuals must at first be paired; in other words, closely in-bred in order that the type of the breed be fixed. Thus the progressive breeder striving to fix certain desired qualities in animals, by rigidly selecting and breeding from only those having the desired qualities in a high degree, will of necessity be driven to mate animals closely related in blood. As a breed increases in numbers there is less necessity for mating closely related animals. By judicious in-breeding animals are rendered more prepotent, but it must never be forgotten that while in-breeding intensifies good qualities it also intensifies bad ones. Prepotency of blood acts strongly when there is a variation toward a defect, as in the direction of a more perfect form. It is the almost universal belief of breeders that a cross between different breeds, or between individuals of the same breed, but of another strain, gives vigor and fertility to the offspring, and, on the other hand, that close in-breeding diminishes vigor and fertility. Even the most strenuous advocates of in-breeding admit that an outcross at perhaps long distant intervals is indispensable. When any wild species becomes rare, close in-breeding will help to exterminate it. A cross between animals of the same species, which differ to some extent, gives vigor and fertility to the offspring, close in-breeding continued for several generations between the nearest relations if these be kept under the same conditions of life almost always leads to decreased size, weakness, and sterility. On the other hand, crossing of forms exposed to slightly different conditions of life, or which have varied, favors the size, vigor, and fertility of the offspring. Change of climate and environment modify the character of a family or breed, and correlated individuals which have lived for a few generations in widely separated districts give to the offspring the good qualities accomplished by using an outcross. Even in-bred animals from the same strain and without admixture of foreign blood will in the hands of different breeders in the course of time unconsciously diverge from the parental type. Some families in the leading breeds of cattle have been bred so long in certain lines that they may be termed sub-breeds. In breeding circles these highly in-bred animals command a much higher price than those which are miscellaneously bred.

*Crossing.* To obtain a desired qualification (not inherent in a breed) crossing is sometimes resorted to. All the best breeders are strongly opposed to this practice, except sometimes among closely allied sub-breeds. Where two pure breeds are crossed the offspring from the first cross are sometimes quite uniform in character, and everything seems simple enough; but when these mongrels are crossed one with another hardly two are alike, and the difficulties of the task become manifest. Yet native breeds have been largely improved by crossing or blending with improved breeds. By continuous successful crossing some improved breeds have encroached upon, modified, and, in some cases, entirely supplanted the native breeds in certain localities. It is vain, however, to attempt to improve a breed above the capabilities of the soil, unless artificial food is supplied. In some breeds of sheep hardihood and ability to withstand storms and live on scanty fare have been sacrificed to size. Sometimes a violent cross is necessary. The bulldog has been crossed on the greyhound to give the latter gameness and endurance. In a breed which, as in this case, has been crossed only once, the physical characters derived from such a cross will naturally become less and less with each successive generation. Crossing breeds gives rise to atavism and apparently reversion to original parent forms having entirely different characters to their immediate ancestors. This is more often the case when cross-bred animals are mated. In these cases reversion to a character of any degree of antiquity may occur. It would appear that when once you break the fixed character of a species or breed variation goes on for a long period and is liable to recur indefinitely, but where a distinct variety or pure breed is used on a cross this tendency becomes weaker in each generation until the tendency is obliterated.

In the foregoing remarks I have endeavored to set before you, imperfectly it is true, the opinions of the ablest scientific investigators as well as practical breeders on what are the leading principles governing the art of breeding. The question in regard to which particular kind of horse, cow, sheep, or pig to breed must of necessity be determined by the breeder himself. Climate and soil, training, and aptitude of the individual breeder for developing to the fullest extent and fixing the best qualities of a particular breed, all these are qualities contributing to success. In order to achieve success there is no necessity for endeavoring to originate a new breed; life is too short to accomplish this, and there are breeds enough already for all purposes. He who endeavors to raise the average of his herd, flock, or stud up to the standard of his ideal, if that standard has been pitched sufficiently high, will have scope enough for his talents as a breeder. This is an age of specialization in the agricultural world, and persistent, well-directed effort in breeding in one particular direction with some clearly defined and desirable object in view will ultimately establish the reputation of the individual as a successful breeder.

